# Investigation of Prothrombin G20210A and *Factor V Leiden* G1691A Variants in Patients with Acute Coronary Syndrome Presenting to the Emergency Department with Chest Pain

**DOI:** 10.3390/genes16121490

**Published:** 2025-12-12

**Authors:** Fulya Yukcu, Murtaza Kaya, Fatmagul Can, Harun Yildirim

**Affiliations:** 1 Department of Biophysics, Faculty of Medicine, Kutahya Health Sciences University, Kutahya 43100, Türkiye; 2 Department of Emergency Medicine, Faculty of Medicine, Kutahya Health Sciences University, Kutahya 43100, Türkiye; murtaza.kaya@ksbu.edu.tr (M.K.); harun.yildirim@ksbu.edu.tr (H.Y.); 3 Department of Medical Biochemistry, Faculty of Medicine, Kutahya Health Sciences University, Kutahya 43100, Türkiye; fatmagul.can@ksbu.edu.tr

**Keywords:** acute coronary syndrome, *factor V Leiden*, prothrombin, genetic variant, logistic regression

## Abstract

Background: Acute coronary syndrome (ACS) is a major cardiovascular emergency influenced by environmental and genetic factors. Thrombophilic variants such as prothrombin G20210A (rs1799963) and *factor V Leiden* G1691A (rs6025) may influence thrombin generation and has been reported to show associations with coronary events. Methods: This case–control study included 100 ACS patients and 131 age and sex-matched healthy controls. Genotyping of rs1799963 and rs6025 was performed using polymerase chain reaction followed by restriction fragment length polymorphism (PCR-RFLP) analysis. Results: The GG genotype was markedly more common among ACS patients for both variants. For rs1799963, carriers of the A allele (GA + AA) were less common in ACS (2.0%) than controls (9.2%; *p* = 0.039), corresponding to an 8.6-fold higher odds of ACS in GG carriers (OR = 8.624; 95% CI: 1.757–42.345; *p* = 0.008). For rs6025, A allele carriers (9.0%) were also reduced in ACS versus controls (18.3%; *p* = 0.049), and GG homozygotes exhibited a 2.6-fold higher risk (OR = 2.635; 95% CI: 1.104–6.290; *p* = 0.029). Age was independently associated with higher ACS risk (OR = 1.047; 95% CI: 1.029–1.066; *p* < 0.001). Conclusions: Our findings indicate that the rs1799963 and rs6025 variants were independently associated with ACS, together with advancing age. Both the GG genotype and older age were associated with higher odds of ACS, whereas A-allele carriers appeared less common among ACS cases.

## 1. Introduction

Acute coronary syndrome (ACS) comprises a range of acute myocardial ischemic conditions resulting from an abrupt, critical, or sub-occlusive reduction in coronary blood flow, most commonly due to atherosclerotic plaque rupture or erosion with subsequent thrombus formation [1,2]. This clinical entity includes unstable angina, non-ST-elevation myocardial infarction (NSTEMI) and ST-elevation myocardial infarction (STEMI), and may also present as dynamic ischemic patterns such as crescendo angina or ischemia triggered by a sub-occlusive coronary thrombus [1,3]. The degree and duration of ischemia determine the extent of myocardial injury, producing a continuum that ranges from reversible supply–demand mismatch to complete transmural infarction [2]. This definition reflects contemporary international cardiology guidelines, which characterize ACS as a group of acute atherothrombotic syndromes sharing common pathophysiological mechanisms despite differing electrocardiographic and biomarker profiles [1,3]. The development of ACS is the result of a complex pathophysiological process involving the progression of atherosclerosis, disruption of endothelial integrity, and an ongoing inflammatory response in the vessel wall. Lipid accumulation, macrophage infiltration, and cytokine release within the atherosclerotic plaque weaken the fibrous cap, thereby creating a susceptible substrate for plaque rupture or endothelial erosion [1,2]. Following these events, platelet activation, initiation of the coagulation cascade and intraluminal thrombus formation lead to partial or complete occlusion of the coronary lumen, thereby shaping the clinical spectrum of ACS [1]. Traditional cardiovascular risk factors such as age, hypertension, diabetes, dyslipidemia and smoking further accelerate this process by enhancing oxidative stress, exacerbating endothelial dysfunction and amplifying the inflammatory response [1,3]. Moreover, recent evidence indicates that genetic susceptibility—particularly polymorphic variants in genes related to coagulation and platelet responsiveness—significantly contributes to individual differences in ACS development by enhancing prothrombotic tendency [4].

Prothrombin G20210A (*F2*, rs1799963) and *factor V Leiden* G1691A (*F5*, rs6025) gene polymorphisms are among the most extensively studied hereditary thrombophilia markers [5]. The *F2* gene, located on chromosome 11p11-q12, encodes prothrombin, a key precursor of thrombin in the coagulation cascade [6]. The rs1799963 polymorphism is characterized by a guanine-to-adenine substitution at position 20210 in the 3′ untranslated region of the *F2* gene. This variant increases plasma prothrombin levels, contributing to a hypercoagulable state and a higher risk of thrombus formation. It has also been associated with increased susceptibility to coronary artery disease and stroke in diverse populations [7,8].

The *F5* gene, located on chromosome 1q23, encodes coagulation *factor V*, a glycoprotein that plays a central role in the blood coagulation cascade by serving as a cofactor for the prothrombinase complex. Upon activation, *factor V* accelerates the conversion of prothrombin to thrombin, thereby facilitating clot formation [9]. Located in exon 10 of the *F5* gene, the rs6025 polymorphism arises from a point mutation at nucleotide 1691, causing an arginine to glutamine substitution at position 506, one of the primary cleavage sites for activated protein C [10].

Both polymorphisms are well-established risk factors for venous thromboembolism (VTE), with numerous studies demonstrating a strong association with deep vein thrombosis and pulmonary embolism. However, their involvement in arterial thrombotic disorders—particularly in ACS—remains controversial. Although some evidence supports a contributory role, especially among younger patients or those without conventional cardiovascular risk factors, phenotype-specific meta-analyses have reported heterogeneous results across populations, underscoring the need for further population-specific studies [11,12,13]. Given the high prevalence and clinical significance of ACS, identifying genetic markers that may contribute to its development could facilitate risk stratification, early diagnosis, and personalized therapeutic approaches. In this context, this study investigates whether rs1799963 and rs6025 gene polymorphisms are associated with ACS in patients presenting to the emergency department with chest pain.

## 2. Materials and Methods

### 2.1. Study Design

This cohort case–control study was conducted between 12 August and 12 September 2025 at the Department of Emergency Medicine, Faculty of Medicine, Kutahya Health Sciences University and the Emergency Department of Kutahya City Hospital. Blood samples were collected from participants and analyzed at the Physiology and Biophysics Laboratory of Kutahya Health Sciences University. The study received ethical approval from the Kutahya Health Sciences University Non-Interventional Clinical Research Ethics Committee (Decision No: 2025/10-07; 11 August 2025), and written informed consent was obtained from each participant prior to data collection.

### 2.2. Patient Selection

This study enrolled individuals aged 18 years and older who presented to the emergency department with complaints of chest pain. Eligibility criteria included evidence of electrocardiographic abnormalities and elevated cardiac biomarkers indicative of an ACS. Patients were excluded if they were under 18 years of age, had a known history of malignancy, coagulation disorders, myocarditis, endocarditis, arrhythmias, or were pregnant, as well as those with hereditary dyslipidemias such as familial hypercholesterolemia (including homozygous forms) or chronic systemic inflammatory diseases, including rheumatoid arthritis. ACS diagnoses were made according to the guidelines of the American College of Cardiology (ACC), American Heart Association (AHA), and the European Society of Cardiology (ESC), which cover USAP, NSTEMI, and STEMI [3,14]. The control group consisted of adults (≥18 years) who presented to the emergency department with chest pain but were not diagnosed with ACS and who had no personal or family history of ACS or atherosclerotic cardiovascular disease

### 2.3. Sample Size

The sample size was determined based on prior data and power analysis using the G*Power software (version 3.1.9.7, Heinrich Heine University Düsseldorf, Germany). A total of 231 participants were enrolled, comprising 100 patients diagnosed with ACS and 131 controls. The statistical power was calculated for detecting an association between genotype distributions and disease status, assuming an effect size (odds ratio) of 2.1 derived from the study by Isordia-Salas et al., with a significance level (α) of 0.05 and an allocation ratio of 0.76 [12]. Under these parameters, the achieved power (1–β) was calculated as 86%, confirming that the sample size was sufficient to detect statistically meaningful differences between the ACS and control groups. This sample framework ensured sufficient sensitivity to evaluate potential associations between prothrombin G20210A and *factor V Leiden* G1691A polymorphisms and ACS risk.

### 2.4. Data Collection

Demographic data, including age and sex, were recorded. Because this study was conducted in the emergency department, only clinical and laboratory parameters routinely obtained at admission could be collected. These included vital signs such as blood pressure and heart rate, along with standard biochemical tests, including glucose and renal and liver function markers. Information on chronic cardiovascular risk factors—such as smoking status, history of hypertension or diabetes, and lipid profile measurements—was not consistently available for all participants and therefore was not included in the analysis. Approximately 2 mL of peripheral blood was drawn into sterile EDTA-coated tubes and stored at −20 °C until genomic DNA extraction.

### 2.5. DNA Extraction and Amplification

Genomic DNA was isolated from peripheral blood samples collected in EDTA tubes using the Monarch Genomic DNA Purification Kit (New England Biolabs, NEB, Ipswich, MA, USA). The rs1799963 and rs6025 gene polymorphisms were evaluated through polymerase chain reaction followed by restriction fragment length polymorphism analysis (PCR-RFLP) [15,16]. The enzymatically digested PCR products were electrophoresed on 2.5% agarose gels (Biomax, HS-8000, Ankara, Türkiye) stained with ethidium bromide (BioShop, ETB444.50, Burlington, ON, Canada) and visualized under ultraviolet (UV) transillumination using a 100 bp DNA marker (abm, G193, Richmond, BC, Canada). Table 1 provides a summary of the primer sequences, PCR protocols, restriction enzymes used and the genotyping outcomes. Genotyping accuracy was ensured by including positive and negative controls in each PCR-RFLP run and by re-analyzing approximately 10% of the samples, which showed complete concordance with the initial results.

### 2.6. Statistical Analysis

All statistical procedures were conducted using Statistical Package for the Social Sciences (SPSS) software version 27.0.1 (IBM Corp., Armonk, NY, USA). Associations were assessed using standard statistical tests. The distribution of continuous variables was assessed via the Kolmogorov–Smirnov normality test. Normally distributed data were shown as the mean ± standard deviation (SD) and analyzed using the Student’s *t*-test. For data not conforming to normal distribution, non-parametric tests such as the Mann–Whitney U tests were applied. Non-normally distributed data are presented as median [IQR 25–75]. The distribution of categorical variables, including genotype frequencies, was compared between groups using chi-square (χ^2^) tests. When the expected frequency in any cell was less than 5, Fisher’s exact test was applied instead. Odds ratios (OR) and 95% confidence intervals (CI) were calculated to determine the association between genotypes, alleles and ACS risk. Hardy–Weinberg equilibrium (HWE) was assessed in the control group using the χ^2^ test. A *p*-value < 0.05 was considered statistically significant. Because the A allele was extremely rare in both variants, a dominant genetic model (GA + AA vs. GG) was applied, which is the standard approach when allele frequencies do not permit separate analysis of minor homozygotes. Additional hierarchical models with extended clinical and biochemical covariates were also constructed and are provided in the Supplementary Materials.

## 3. Results

### 3.1. Demographic, Clinical, and Biochemical Characteristics of the Study Groups

The demographic, clinical, and biochemical characteristics of the study groups are summarized in Table 2. The mean age of patients with ACS was significantly higher than that of the control group (62.4 ± 13.7 vs. 50.2 ± 18.8 years, respectively; *p* < 0.001). The sex distribution did not differ significantly between groups (*p* = 0.805). There were no statistically significant differences in systolic blood pressure (SBP) or diastolic blood pressure (DBP), or heart rate values (*p* > 0.05). The mean body mass index (BMI) was slightly but significantly higher in the ACS group compared with controls (27.1 ± 5.1 kg/m^2^ vs. 25.7 ± 4.8 kg/m^2^, respectively; *p* = 0.040). Regarding biochemical parameters, glucose, urea, potassium, alanine aminotransferase (ALT), and aspartate aminotransferase (AST) levels were significantly elevated in ACS patients (*p* < 0.05 for all). In contrast, creatinine and sodium levels did not differ significantly between the groups (*p* > 0.05). As expected, serum troponin I levels were markedly higher in the ACS group, with 71% of patients showing values > 100 ng/L, compared to only 14.5% in the control group (*p* < 0.001).

### 3.2. Genotype and Allele Distributions of rs1799963 and rs6025 Polymorphisms

The genotype and allele distributions of the rs1799963 and rs6025 polymorphisms in patients with ACS and controls are presented in Table 3. For the rs1799963 variant, the GG genotype was predominant in both groups but was more frequent among ACS patients compared with controls (98.0% vs. 90.8%, respectively). The GA genotype was observed in 8.4% of controls and 2.0% of patients, while the AA genotype was detected in one control and absent in the ACS group. Under the dominant model (GA + AA vs. GG), carriers of the A allele exhibited a significantly lower frequency in the ACS group (*p* = 0.039). The G allele frequency was higher among patients than controls (99.0% vs. 95.0%, respectively; *p* = 0.032). Genotype distributions were in Hardy–Weinberg equilibrium in the control group (*p* = 0.209).

For the rs6025 polymorphism, the GG genotype was also more common in ACS patients than in controls (91.0% vs. 81.7%, respectively). The GA and AA genotypes were less frequent in patients (8.0% and 1.0%, respectively) compared with controls (16.0% and 2.3%). The dominant model (GA + AA vs. GG) revealed a significantly lower prevalence of A allele carriers among ACS patients (*p* = 0.049). Similarly, the G allele was significantly more frequent in the ACS group (95.0% vs. 89.7%, respectively; *p* = 0.041). The genotype distribution conformed to HWE in the control group (*p* = 0.128).

### 3.3. Association of rs1799963 and rs6025 Genotypes with Clinical and Biochemical Parameters in the Control Group

The relationship between rs1799963 and rs6025 genotypes and clinical and biochemical parameters in the control group is presented in Table 4. For the rs1799963 polymorphism, carriers of the A allele (GA + AA) showed higher mean age values compared with the GG genotype (58.91 ± 22.35 vs. 49.28 ± 18.34 years, *p* = 0.092), although the difference did not reach statistical significance. No significant differences were observed between genotypes with respect to sex distribution, blood pressure (SBP, DBP), heart rate, BMI, or most biochemical parameters (*p* > 0.05). However, the mean glucose level was significantly higher among A allele carriers than among GG homozygotes (168.33 ± 102.12 vs. 119.7 ± 59.95 mg/dL, *p* = 0.014). Levels of urea, creatinine, sodium, potassium, ALT, AST, and troponin I did not differ significantly between genotypes (*p* > 0.05).

For the rs6025 polymorphism, there were no statistically significant differences between GG and GA + AA genotypes regarding demographic or biochemical parameters. Although A allele carriers tended to have slightly higher DBP and potassium levels, these differences were not statistically significant (*p* = 0.108 and *p* = 0.074, respectively).

### 3.4. Association of rs1799963 and rs6025 Genotypes with Clinical and Biochemical Parameters in the ACS Group

The association between rs1799963 and rs6025 genotypes and clinical and biochemical parameters in patients with ACS is summarized in Table 5. For the rs1799963 polymorphism, carriers of the A allele (GA + AA) showed comparable age, blood pressure, heart rate, and BMI values to those with the GG genotype (*p* > 0.05). No significant differences were observed in glucose, urea, creatinine, sodium, potassium, ALT, AST, or troponin I levels between genotypes (*p* > 0.05). All carriers of the A allele were male and exclusively presented with unstable angina pectoris (USAP), with no cases of STEMI or NSTEMI, indicating a statistically significant distribution (*p* = 0.001).

For the rs6025 polymorphism, no statistically significant differences were found between GG and GA + AA genotypes in terms of demographic or biochemical parameters (*p* > 0.05). Importantly, the presence of the A allele (GA + AA genotypes) was significantly more frequent among patients with STEMI compared with those with NSTEMI or USAP (*p* = 0.049).

### 3.5. Logistic Regression Analysis of Independent Predictors for ACS

Binomial logistic regression analysis was performed to identify independent predictors associated with ACS. The overall model was statistically significant (χ^2^ = 42.1, *p* < 0.001), explaining approximately 22.3% of the variance in ACS occurrence (Nagelkerke R^2^ = 0.223) and demonstrating a good model fit (AIC = 284, Deviance = 274).

Among the variables included in the model, age, rs1799963, and rs6025 genotypes were identified as significant independent predictors of ACS (*p* < 0.05). Each one-year increase in age was associated with a 1.047-fold higher risk of ACS (OR = 1.047, 95% CI: 1.029–1.066). Carriers of the rs1799963 GG genotype showed approximately 8.6-fold higher odds of being in the ACS group (OR = 8.624, 95% CI: 1.757–42.345, *p* = 0.008). Similarly, the GG genotype of rs6025 was associated with a 2.6-fold higher risk (OR = 2.635, 95% CI: 1.104–6.290, *p* = 0.029). In contrast, sex was not a significant predictor in the model (*p* = 0.822).

**Figure 1:**
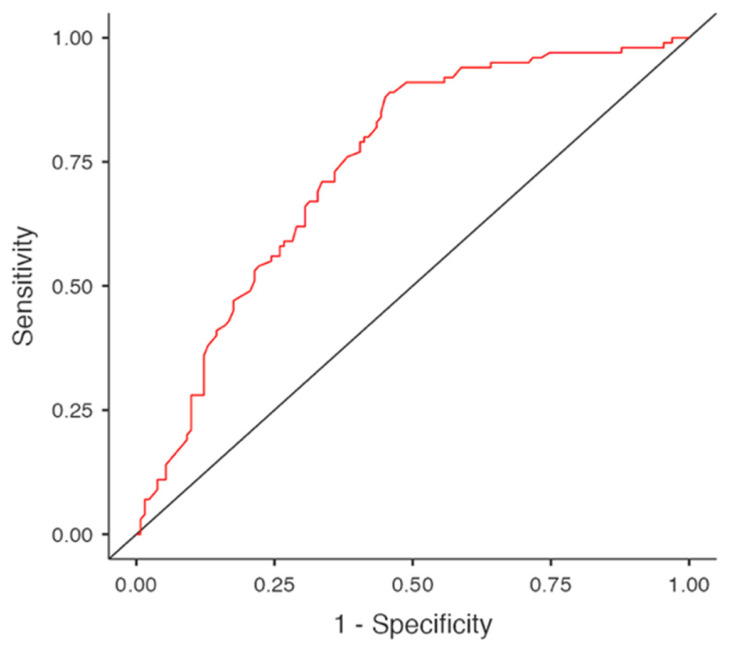
ROC curve of the logistic regression model (AUC = 0.743). ROC, Receiver Operating Characteristic; AUC, Area Under the Curve.

The discriminative ability of the regression model was further supported by the Receiver Operating Characteristic (ROC) curve analysis (AUC = 0.743), which demonstrated moderate discrimination ability in distinguishing ACS cases from controls and was consistent with the observed explanatory power of the model (Figure 1).

The extended multivariable models (Model 2 and Model 3), which incorporated additional clinical and biochemical variables, are presented in Supplementary Tables S1 and S2.

## 4. Discussion

ACS is a multifactorial clinical condition, resulting from the combined influence of environmental risk factors and inherited genetic predispositions—particularly those influencing thrombotic balance [17]. Among inherited thrombophilic markers, the rs1799963 and rs6025 polymorphisms have been extensively studied due to their functional roles in enhancing thrombin generation and conferring resistance to activated protein C, respectively. These variants have been consistently reported to show strong associations with venous thromboembolism, and several studies have also documented their association with arterial thrombotic events—including myocardial infarction—particularly in younger individuals [11,12]. However, while the association between these mutations and venous events is well-established, their contribution to arterial thrombosis, particularly ACS, remains controversial, with studies yielding inconsistent findings across different populations [7,11]. In this context, the present study aimed to investigate the potential association of the rs1799963 and rs6025 variants with susceptibility to ACS by comparing genotype and allele distributions between patients with ACS and controls.

Our study revealed several significant clinical and biochemical differences between ACS patients and controls. The mean age of ACS patients was significantly higher than that of the control group (62.4 ± 13.7 vs. 50.2 ± 18.8 years, *p* < 0.001), which aligns with existing literature highlighting age as a major non-modifiable risk factor for ACS. Age-related endothelial dysfunction, progressive atherosclerotic changes, and reduced vascular resilience have all been implicated in the increased susceptibility to coronary events among older individuals [14]. Similarly, large-scale epidemiological studies, including the 2023 ESC guidelines, have demonstrated that ACS incidence increases sharply with advancing age [1]. Additionally, BMI was significantly elevated in ACS patients (27.1 ± 5.1 kg/m^2^ vs. 25.7 ± 4.8 kg/m^2^, *p* = 0.040), consistent with the well-established contribution of excess adiposity to metabolic dysregulation and systemic inflammation, which promote atherothrombotic processes [18].

Notably, glucose levels were significantly higher in the ACS group (median 134 mg/dL vs. 105 mg/dL, *p* < 0.001), which aligns with previous studies identifying hyperglycemia as both an independent risk factor and a prognostic indicator in acute coronary events—even among non-diabetic individuals [19]. In our study, urea levels were significantly higher among ACS patients than controls (*p* = 0.004), suggesting possible renal hypoperfusion or neurohormonal activation during acute ischemia. This finding is consistent with previous research demonstrating that elevated blood urea nitrogen (BUN) levels at admission are independently associated with increased in-hospital mortality in ACS patients, highlighting the prognostic significance of renal function markers in the acute phase [20]. Similarly, modest but statistically significant elevations in potassium, ALT, and AST levels were observed. These alterations may reflect cellular injury, hepatic hypoperfusion, or neurohormonal activation occurring during acute coronary events. Most notably, troponin I levels >100 ng/L were present in 71% of ACS patients but only 14.5% of controls (*p* < 0.001), further confirming its diagnostic utility as a sensitive biomarker of myocardial injury [2]. Taken together, these findings underscore the multifaceted clinical and biochemical profile of ACS patients and support the pathophysiological interplay between cardiovascular, renal, hepatic, and metabolic systems during acute coronary events.

In our study, allele frequency analysis of the rs1799963 polymorphism showed a significantly higher prevalence of the G allele in ACS patients compared with controls (99.0% vs. 95.0%, *p* = 0.032), indicating that this allele may contribute to increased susceptibility to ACS. This observation is consistent with the findings of Bădescu et al., who reported a higher frequency of the G allele among myocardial infarction patients harboring inherited thrombophilic variants, particularly prothrombin G20210A and *factor V Leiden* mutations [11]. Similarly, Isordia-Salas et al. demonstrated a significant association between the rs1799963 G allele and ST-elevation myocardial infarction in young Mexican patients [12]. Regarding the rs1799963 polymorphism, no significant difference in individual genotype frequencies was observed between ACS patients and controls. However, when evaluated under the dominant model (GA + AA vs. GG), carriers of the A allele exhibited a significantly lower frequency in the ACS group (*p* = 0.039), suggesting a possible protective effect of the A allele against disease development. The genotype distribution for this variant conformed to Hardy–Weinberg equilibrium in the control group (*p* = 0.209). The lack of statistical significance in the genotype-based comparison may be attributed to the limited number of GA and AA carriers, which reduced the statistical power of the analysis. Nevertheless, this trend is consistent with previous reports indicating a potential role of the rs1799963 variant in modulating thrombotic risk and ACS susceptibility [11,12]. In contrast, Shibeeb et al. observed a significantly higher GA genotype frequency among ACS patients in a Middle Eastern population, highlighting the potential influence of ethnic and genetic background on the distribution and clinical impact of this polymorphism [10].

For the rs6025 polymorphism, no significant difference was observed in the distribution of individual genotypes between ACS patients and controls. However, when analyzed under the dominant genetic model (GA + AA vs. GG), A allele carriers were significantly less frequent among ACS patients (*p* = 0.049). In parallel, the G allele frequency was higher in the ACS group compared with controls (95.0% vs. 89.7%, *p* = 0.041). These findings suggest that the A allele may not uniformly increase thrombotic risk and could potentially exert a partial protective effect against ACS development under certain environmental or genetic contexts. The genotype distribution for this variant conformed to HWE in the control group (*p* = 0.128).

A large number of well-designed studies have confirmed that the A allele of the rs6025 variant is an important genetic risk factor for VTE. This association appears to be even stronger in several populations, including European, Middle Eastern, and Qatari cohorts [10,21,22,23]. In contrast to VTE, the relationship between the A allele and arterial events such as MI and ACS has been inconsistent [24,25]. Large biobank studies have reported no significant association between the A allele and arterial thrombosis, including MI and stroke [24]. Additionally, some population-specific studies have found no difference in A-allele frequency between ACS patients and controls, and in some cases even reported a higher frequency among controls [25].

According to large population datasets, both variants examined in this study exhibited low A-allele frequencies in the general population. The rs1799963 A allele is typically observed at ~1–3% in European cohorts and below 2% in Middle Eastern populations, while the rs6025 A allele shows frequencies of ~2–5% in Europeans and ~1–3% in Middle Eastern groups. These published data indicate that low numbers of A-allele carriers are expected in populations similar to ours, providing context for the limited GA/AA genotypes observed in the present study [10,22,23,24].

Our results demonstrated a higher frequency of the A allele in controls (10.3%) than in ACS patients (5.0%), suggesting a potentially protective effect, particularly in heterozygous carriers. Similar findings were reported by Franco et al., who noted that environmental modifiers such as smoking may influence the expression of rs6025-related thrombotic risk, potentially mitigating the pathogenic impact of the A allele in certain populations [17]. In contrast, Badescu et al. reported a higher prevalence of both the A allele and GA genotype among myocardial infarction patients [11]. These conflicting results underscore the population-specific nature of rs6025-associated thrombotic risk, which may be shaped by ethnic background, environmental exposures, and clinical comorbidities. Therefore, the significantly higher frequencies of the GG genotype and G allele in the ACS group underscore a possible role of rs6025 in predisposing individuals to acute coronary events. However, the lower frequency of A allele carriers among ACS patients raises the hypothesis that heterozygosity may not uniformly increase risk and could even confer partial protection under certain conditions.

In the control group, rs1799963 and rs6025 genotypes were not significantly associated with age, sex, BMI, blood pressure, urea, ALT, AST, or other biochemical parameters (*p* > 0.05). However, carriers of the A allele of rs1799963 showed significantly higher glucose levels compared with GG homozygotes (*p* = 0.014). These findings are consistent with results from large population-based cohorts, such as the Qatar Biobank study, which demonstrated that these thrombophilic variants are more prevalent among individuals with a history of thrombotic events but do not correlate with metabolic or biochemical markers in otherwise healthy populations [10]. Similarly, Topoleanu et al. observed no significant impact of these polymorphisms on routine laboratory parameters among controls [26]. Overall, our results suggest that rs1799963 and rs6025 do not exert measurable effects on routine biochemical or clinical parameters in controls, apart from a mild association between rs1799963 and glucose levels that warrants further investigation.

In the ACS group, clinical and biochemical parameters—including age, sex, BMI, blood pressure, heart rate, glucose, renal and hepatic markers—did not differ significantly across rs1799963 and rs6025 genotypes (*p* > 0.05). However, subgroup analysis by clinical presentation revealed genotype-specific patterns under the dominant model. Carriers of the A allele of rs1799963 (GA + AA) were detected exclusively among patients with USAP, with no carriers observed in those with STEMI or NSTEMI (*p* = 0.001). Similarly, A allele carriers of rs6025 were identified only in patients presenting with STEMI and were absent in other ACS subtypes (*p* = 0.049). These findings suggest a potential clinical heterogeneity in the distribution of thrombophilic variants, possibly reflecting differing thrombotic mechanisms across ACS subtypes. Our observation that rs6025 A allele carriers were identified exclusively among patients with STEMI may reflect population-specific differences in the expression of thrombophilic variants. While Mahmoodi et al. found that the rs6025 mutation did not significantly distinguish between STEMI and NSTEMI presentations in a large European cohort, our results suggest that subtle genotype-dependent tendencies might still contribute to the clinical heterogeneity of ACS subtypes in smaller or ethnically distinct populations [27].

In the logistic regression model, age and both rs1799963 and rs6025 genotypes were identified as independent predictors of acute coronary syndrome (ACS), whereas sex did not show a significant association (*p* = 0.822). Each one-year increase in age conferred a 1.047-fold higher risk of ACS (95% CI: 1.029–1.066), consistent with previous population-based analyses showing age as the strongest non-modifiable determinant of coronary events [3,14].

Both rs1799963 and rs6025 genotypes were identified as independent predictors of ACS after adjustment for age and conventional cardiovascular risk factors. Carriers of the rs1799963 GG genotype exhibited an 8.6-fold higher risk of ACS (OR = 8.624, 95% CI: 1.757–42.345, *p* = 0.008), while the rs6025 GG genotype conferred a 2.6-fold increase in risk (OR = 2.635, 95% CI: 1.104–6.290, *p* = 0.029). Together with the ROC curve results (Figure 1), the logistic regression analysis summarized in Table 6 demonstrates the robustness of the model, confirming that genetic and demographic variables jointly contribute to the discrimination of ACS cases from controls. This model suggests that the contribution of both variants to ACS susceptibility may be masked in univariate analyses but becomes evident when potential modifiers are considered. These findings also suggest that both variants may contribute to a prothrombotic state through related mechanisms within the coagulation cascade, potentially enhancing thrombin generation and predisposing to thrombosis beyond conventional risk factors. Consistent with these observations, Rallidis et al. reported that the rs1799963 and rs6025 variants served as independent prothrombotic determinants in very early onset STEMI, and Burzotta et al. demonstrated that their effects on coronary events remained significant in multivariate analyses, particularly among patients with a lower atherosclerotic burden [13,28]. Likewise, Mannucci et al. reported that the rs6025 variant was independently associated with myocardial infarction risk in younger individuals, although the direction of allelic association differed from that observed in the present study [29]. This discrepancy may reflect population-specific genetic backgrounds or differences in allele frequencies, but both studies support the role of inherited thrombophilia as an independent determinant of premature coronary disease.

It is important to note that the multivariate model used in this study was designed to evaluate associations rather than to provide predictive performance. Given the multifactorial nature of ACS, logistic regression models that include only a limited number of clinical and genetic variables typically yield modest explanatory power, a pattern that has also been reported in previous studies [28,29].

However, it should be remembered that atherosclerosis lies at the core of ACS, and that coronary thrombosis represents the final step in most cases, occurring after plaque rupture or endothelial erosion. Since our study was conducted in patients presenting to the emergency department, chronic risk factors that shape atherosclerosis—such as smoking status, histories of hypertension and diabetes, and lipid profiles—could not be systematically recorded for all participants. In addition, the fact that the ACS group was older than the control group is an important factor that may influence the interpretation of the findings, given the strong determining effect of age on the development of atherosclerosis. Therefore, the possible impact of the genetic variants on ACS should be evaluated within the context of the atherosclerotic process and major baseline risk factors, particularly as a complementary component contributing to the thrombotic endpoint.

Taken together, these results indicate that, independent of sex, advancing age and the presence of the GG genotypes of both rs1799963 and rs6025 constitute significant risk factors for ACS, whereas carriers of the GA or AA genotypes appear to show a protective effect. These findings highlight the potential role of genetic variability within the prothrombin and *factor V* loci in modulating individual susceptibility to ACS.

## 5. Limitations

This study has several limitations. The sample size was moderate, and the low frequency of the A alleles of both rs1799963 and rs6025 in our study population naturally limited the number of GA/AA carriers and resulted in wider confidence intervals for the OR estimates. This pattern is commonly observed in studies evaluating low-frequency genetic variants and reflects the genetic characteristics of the sampled population rather than a methodological issue; therefore, these associations should be interpreted with appropriate caution and further validated in larger cohorts. Because the study was conducted in the emergency department, chronic cardiovascular risk factors that influence atherosclerotic burden, such as smoking status, histories of hypertension or diabetes, and lipid profiles, could not be systematically recorded for all participants. As a result, the AUC value derived from the ROC analysis may be affected by the absence of these classical cardiovascular risk factors in the model, which represents an important methodological limitation. The age difference between the ACS and control groups may also have influenced the observed associations. In addition, the investigation focused on two single nucleotide polymorphisms in the prothrombin and *factor V* genes and no functional assays were performed to assess their biological effects on coagulation activity. Future multicenter studies with larger cohorts, comprehensive cardiovascular risk profiling, and broader genetic screening are needed to validate and expand these findings.

## 6. Conclusions

In conclusion, this study suggests that both rs1799963 and rs6025 polymorphisms are associated with ACS risk, independent of age in our regression model. The GG genotypes were identified as potential risk-enhancing variants, whereas the GA and AA genotypes showed a tendency toward a protective effect. These findings highlight a possible contribution of inherited thrombophilic variants to the thrombotic component of ACS, although further studies with comprehensive cardiovascular risk profiling are warranted to confirm these associations.

## Figures and Tables

**Table 1:** Experimental Conditions for the Analysis of Prothrombin G20210A and *Factor V Leiden* G1691A Variants.

Genetic Variants	Prime Sequence (5′-3′)	Tm (°C)	PCR Product Size	Restriction Enzyme	Genotyping (bp)
Prothrombin G20210A (rs1799963)	F-ATG GGG TGA AGG CTG TGA CC R-AGC ACT GGG AGC ATT GAG CCT	60 °C	221 bp	MnII	GG: 221 GA: 221–192 AA: 192
*Factor V* *Leiden* G1691A (rs6025)	F-TCA GGC AGG AAC AAC ACC AT R-GGT TAC TTC AAG GAC AAA ATA CCT GTA AAG CT	58 °C	241 bp	HindIII	GG: 241 GA: 241–209 AA: 209

Tm, Primer Annealing Temperature; PCR, Polymerase Chain Reaction; bp, Base Pairs.

**Table 2:** Comparison of demographic, clinical, and biochemical characteristics between patients with ACS and controls.

Characteristics	Groups		*p*-Value
	Control (n = 131)	ACS (n = 100)	
Age	50.2 ± 18.8	62.4 ± 13.7	<0.001 ^a^
Sex			
Female [n (%)]	49 (37.4%)	39 (39.0%)	0.805 ^b^
Male [n (%)]	82 (62.6%)	61 (61.0%)	
SBP (mm/Hg)	127.7 ± 21.7	125.7 ± 22.9	0.495 ^a^
DBP (mm/Hg)	79.1 ± 14.9	80.9 ± 14.8	0.359 ^a^
Heart rate (per/min)	91.3 ± 19.5	87.9 ± 14.3	0.143 ^a^
BMI (kg/m^2^)	25.7 ± 4.8	27.1 ± 5.1	0.040 ^a^
Glucose (mg/dL)	105 (90–127)	134 (109–182)	<0.001 ^c^
Urea (mg/dL)	28.8 (23.6–39.1)	34.6 (26.6–45.9)	0.004 ^c^
Creatinine (mg/dL)	0.87 (0.71–1.06)	0.93 (0.75–1.09)	0.242 ^c^
Sodium (mmol/L)	139 (137–140)	138 (136–140)	0.063 ^c^
Potasyum (mmol/L)	4.20 (3.90–4.60)	4.35 (4.00–4.73)	0.041 ^c^
ALT (U/L)	17 (12–25)	22 (15–33)	0.002 ^c^
AST (U/L)	21 (17–28)	31 (23–46)	<0.001 ^c^
Troponin I (ng/L)			
<100 [n (%)]	112 (85.5%)	29 (29%)	<0.001 ^b^
>100 [n (%)]	19 (14.5%)	71 (71%)	

^a^: Student *t* test, mean ± SD; ^b^: χ^2^ test, noun (%); ^c^: Mann–Whitney U, median IQR 25–75; ALT, Alanine aminotransferase; AST, Aspartate Aminotransferase; BMI, Body mass index; ACS, Acute Coronary Syndrome.

**Table 3:** Genotype and allele distributions of rs1799963 and rs6025 polymorphisms in patients with ACS and controls.

Genetic Variants	Genotype/Allele	Control (n = 131)	ACS (n = 100)	OR (95% CI)	*p* Value	HWE *p* (Control)
rs1799963	GG	119 (90.8%)	98 (98.0%)	4.52 (0.98–20.92)	0.053	—
	GA	11 (8.4%)	2 (2.0%)	1.00 (reference)	-	—
	AA	1 (0.8%)	0 (0.0%)	1.53 (0.04–49.80)	0.810	—
	Dominant (AA + GA vs. GG)	12 (9.2%)	2 (2%)	4.94 (1.08–22.60)	0.039	0.209
	G allele	249 (95.0%)	198 (99.0%)	5.17 (1.15–23.17)	0.032	—
	A allele	13 (5.0%)	2 (1.0%)	1.00 (reference)	—	—
rs6025	GG	107 (81.7%)	91 (91.0%)	2.23 (0.94–5.28)	0.068	—
	GA	21 (16.0%)	8 (8.0%)	1.00 (reference)	-	—
	AA	3 (2.3%)	1 (1.0%)	0.87 (0.08–9.69)	0.913	—
	Dominant (AA + GA vs. GG)	24 (18.3%)	9 (9.0%)	2.26 (1.00–5.12)	0.049	0.128
	G allele	235(89.7%)	190 (95.0%)	2.18 (1.03–4.62)	0.041	—
	A allele	27 (10.3%)	10 (5.0%)	1.00 (reference)	—	—

Significance between groups: *p* < 0.05. Analysis by chi-square (χ^2^), n (%). OR, odds ratio; CI, confidence interval; HWE, Hardy–Weinberg equilibrium; ACS, Acute Coronary Syndrome.

**Table 4:** Association between rs1799963 and rs6025 genotypes and demographic, clinical, and biochemical parameters in the control group.

Characteristic	rs1799963 Genotypes		*p*-Value	rs6025 Genotypes		*p*-Value
	GG (119)	GA + AA(12)		GG(107)	GA + AA(24)	
Age	49.28 ± 18.34	58.91 ± 22.35	0.092 ^a^	49.76 ± 19.58	51.83 ± 15.42	0.632 ^a^
Sex						
Female [n (%)]	44 (37.0%)	5 (41.7%)	0.749 ^b^	38 (35.5%)	11 (45.8%)	0.345 ^b^
Male [n(%)]	75 (63.0%)	7 (58.3%)		69 (64.5%)	13 (54.2%)	
SBP (mm/Hg)	127.63 ± 21.77	128.75 ± 22.02	0.866 ^a^	127.48 ± 22.28	128.87 ± 19.37	0.777 ^a^
DBP (mm/Hg)	79.07 ± 14.94	79.0 ± 15.63	0.988 ^a^	78.07 ± 13.44	83.50 ± 20.13	0.108 ^a^
Heart rate (per/min)	91.10 ± 19.58	93.75 ± 19.20	0.655 ^a^	92.41 ± 19.60	86.58 ± 18.55	0.186 ^a^
BMI (kg/m^2^)	25.61 ± 4.84	26.50 ± 5.21	0.546 ^a^	25.57 ± 4.91	26.25 ± 4.68	0.534 ^a^
Glucose (mg/dL)	119.7 ± 59.95	168.33 ± 102.12	0.014 ^a^	124.20 ± 66.55	124.29 ± 64.28	0.995 ^a^
Urea (mg/dL)	35.16 ± 21.61	33.66 ± 12.73	0.814 ^a^	35.63 ± 21.74	32.32 ± 16.90	0.488 ^a^
Creatinine (mg/dL)	1.04 ± 0.86	0.79 ± 0.17	0.339 ^a^	1.03 ± 0.89	0.97 ± 0.41	0.804 ^a^
Sodium (mmol/L)	138.67 ± 2.21	137.83 ± 6.67	0.337 ^a^	138.56 ± 3.01	138.75 ± 2.17	0.772 ^a^
Potassium (mmol/L)	4.27 ± 0.52	4.28 ± 0.57	0.916 ^a^	4.23 ± 0.49	4.44 ± 0.65	0.074 ^a^
ALT (U/L)	17.0 (12.0–25.5)	13.5 (9.8–20.3)	0.248 ^c^	16.0 (11.5–24.0)	19.0 (15.5–26.0)	0.314 ^c^
AST (U/L)	21.0 (21.0–27.5)	20.0 (14.8–28.0)	0.573 ^c^	21.0 (21.0–27.0)	21.0 (16.0–32.5)	0.425 ^c^
Troponin I (ng/L)						
<100 [n (%)]	101 (84.9%)	11 (91.7%)	1.00 ^b^	90 (84.1%)	22 (91.7%)	0.524 ^b^
>100 [n (%)]	18 (15.1%)	1 (8.3%)		17 (15.9%)	2 (8.3%)	

^a^: Student *t* test, mean ± SD; ^b^: χ^2^ test, noun (%); ^c^: Mann–Whitney U, median IQR 25–75; mean (±SD). ALT, Alanine aminotransferase; AST, Aspartate Aminotransferase; SBP, Systolic blood pressure; DBP, Diastolic blood pressure; BMI, Body mass index.

**Table 5:** Association between rs1799963 and rs6025 genotypes and demographic, clinical, and biochemical parameters in the ACS group.

Characteristic	rs1799963 Genotypes		*p*-Value	rs6025 Genotypes		*p*-Value
	GG (98)	GA + AA (2)		GG (91)	GA + AA (9)	
Age	62.45 ± 13.80	61.50 ± 7.78	0.923 ^a^	62.66 ± 13.22	60.11 ± 18.55	0.597 ^a^
Sex						
Female [n (%)]	39 (39.8%)	0 (0%)	0.519 ^b^	34 (37.4%)	5 (55.6%)	0.306 ^b^
Male [n (%)]	59 (60.2%)	2 (100%)		57 (62.6%)	4 (44.4%)	
SBP (mm/Hg)	125.47 ± 22.93	137.50 ± 30.41	0.466 ^a^	125.10 ± 22.80	131.89 ± 25.17	0.400 ^a^
DBP (mm/Hg)	80.90 ± 14.97	80.00 ± 4.24	0.933 ^a^	80.74 ± 14.51	82.33 ± 18.75	0.760 ^a^
Heart rate (per/min)	87.67 ± 14.23	101.0 ± 14.14	0.193 ^a^	88.53 ± 14.35	82.00 ± 12.57	0.192 ^a^
BMI (kg/m^2^)	27.09 ± 5.14	25.26 ± 5.07	0.620 ^a^	27.01 ± 5.16	27.47 ± 4.96	0.797 ^a^
Glucose (mg/dL)	158.69 ± 75.66	99.00 ± 29.69	0.270 ^a^	158.71 ± 78.35	145.22 ± 33.62	0.611 ^a^
Urea (mg/dL)	41.00 ± 24.58	33.25 ± 17.60	0.659 ^a^	41.89 ± 25.16	30.25 ± 10.73	0.174 ^a^
Creatinine (mg/dL)	1.03 ± 0.52	0.98 ± 0.15	0.891 ^a^	1.05 ± 0.54	0.84 ± 0.21	0.265 ^a^
Sodium (mmol/L)	135.58 ± 18.06	138.50 ± 2.12	0.821 ^a^	135.66 ± 18.73	135.44 ± 3.01	0.973 ^a^
Potassium (mmol/L)	4.43 ± 0.58	4.30 ± 0.14	0.761 ^a^	4.42 ± 0.54	4.46 ± 0.89	0.818 ^a^
ALT (U/L)	22.0 (15.0–32.8)	52.5 (36.8–68.3)	0.337 ^c^	22.0 (15.0–34.5)	24.0 (13.0–30.0)	0.695 ^c^
AST (U/L)	30.05 (22.3–47.3)	33.5 (28.3–38.8)	1.000 ^c^	31.0 (23.0–46.5)	27.0 (21.0–31.0)	0.332 ^c^
Troponin I (ng/L)						
<100 [n (%)]	27 (27.6%)	2 (100%)	0.082 ^b^	27 (29.7%)	2 (22.2%)	0.239 ^b^
>100 [n (%)]	71 (72.4%)	0 (0.0%)		64 (70.3%)	7 (77.8%)	
Diagnosis						
STEMI [n (%)]	17 (17.3%)	0(0%)	0.001 ^b^	13 (14.3%)	4 (44.4%)	0.049 ^b^
NSTEMI [n (%)]	70 (71.4%)	0(0%)		66 (72.5%)	4 (44.4%)	
USAP [n (%)]	11 (11.3)	2 (100%)		12 (13.2)	1 (11.2%)	

^a^: Student *t* test, mean ± SD; ^b^: χ^2^ test, noun (%); ^c^: Mann–Whitney U, median IQR 25–75; mean (±SD). ACS, Acute Coronary Syndrome; ALT, Alanine aminotransferase; AST, Aspartate Aminotransferase; STEMI, ST-Elevation Myocardial Infarction; NSTEMI, Non-ST Elevation Myocardial Infarction; USAP, unstable angina pectoris; SBP, Systolic blood pressure; DBP, Diastolic blood pressure; BMI, Body mass index.

**Table 6:** Binomial logistic regression analysis of independent predictors associated with ACS.

Predictor	B	SE	Z	*p* Value	OR (95% CI)
Intercept	−5.758	1.285	−5.103	<0.001	0.003 (3.46–0.02)
Age	0.046	0.009	5.091	<0.001	1.047 (1.029–1.066)
rs1799963 (GG/GA + AA)	2.154	0.811	2.654	0.008	8.624 (1.757–42.345)
rs6025 (GG/GA + AA)	0.969	0.443	2.183	0.029	2.635 (1.104–6.290)
Sex (Male–Female)	−0.067	0.301	−0.225	0.822	0.934 (0.517–1.688)

Overall model test: χ^2^ = 42.1, *p* < 0.001, R^2^N = 0.223, AIC = 284, Deviance = 274. B, Regression coefficient; SE, Standard error; Z, Wald test statistic; OR, Odds ratio; CI, Confidence interval; R^2^N, Nagelkerke R-squared; AIC, Akaike information criterion; ACS, Acute coronary syndrome.

## Data Availability

The original contributions presented in this study are included in the article/Supplementary Materials. Further inquiries can be directed to the corresponding author.

## Supplementary Materials

The following supporting information can be downloaded at: https://www.mdpi.com/article/10.3390/genes16121490/s1, Table S1: Binomial Logistic Regression Model 2; Table S2: Binomial Logistic Regression Model 3.

## Abbreviations

ACS	Acute Coronary Syndrome
AHA	American Heart Association
ALT	Alanine Aminotransferase
AST	Aspartate Aminotransferase
AUC	Area Under the Curve
BMI	Body Mass Index
BUN	Blood Urea Nitrogen
CI	Confidence Interval
DNA	Deoxyribonucleic Acid
EDTA	Ethylenediaminetetraacetic Acid
ESC	European Society of Cardiology
F2	Prothrombin Gene
F5	Factor V Gene
HWE	Hardy–Weinberg Equilibrium
NSTEMI	Non-ST-Elevation Myocardial Infarction
OR	Odds Ratio
PCR-RFLP	Polymerase Chain Reaction-Restriction Fragment Length Polymorphism
ROC	Receiver Operating Characteristic
SBP	Systolic Blood Pressure
DBP	Diastolic Blood Pressure
SD	Standard Deviation
SNP	Single Nucleotide Polymorphism
SPSS	Statistical Package for the Social Sciences
STEMI	ST-Elevation Myocardial Infarction
USAP	Unstable Angina Pectoris
UV	Ultraviolet
VTE	Venous Thromboembolism
