# Supplementary material for: Investigation of Prothrombin G20210A and Factor V Leiden G1691A Variants in Patients with Acute Coronary Syndrome Presenting to the Emergency Department with Chest Pain
Source: Genes (Basel). 2025 Dec 12;16(12):1490. doi: 10.3390/genes16121490 (PMC12733124; doi:10.3390/genes16121490)
Supplement: Supplementary file 1 [file genes-16-01490-s001.zip › Supplement Table S1.pdf]

Binomial Logistic Regression Model 2

Model Fit Measures

				Overall Model Test		
**Model**	**Deviance**	**AIC**	**R^2N**	**χ^2**	**df**	***p***
1	266	284	0.260	49.8	8	**<.001**

Models estimated using sample size of N = 231.

Model Coefficients – groups

					95% Confidence Interval
**Predictor**	**B**	**SE**	**Z**	***p* value**	**OR (95% CI)**
**Intercept**	-0.560211	1.97050	-2.843	**0.004**	0.000369 (7.765 – 0.176)
**Age**	0.04033	0.00961	4.198	**<.001**	1.04116 (1.022 – 1.061)
**rs1799963 (GG/GA +AA)**	2.06641	0.86429	2.391	**0.017**	7.89643 (1.451 – 42.965)
**rs6025 (GG/GA +AA)**	0.90295	0.44470	2.030	**0.042**	2.46688 (1.032 – 5.898)
**Sex (Male–Female)**	0.12822	0.31086	0.412	0.680	1.13681 (0.618 – 2.091)
**SBP**	-0.00864	0.00676	-1.279	0.201	0.99140 (0.978 – 1.005)
**BMI**	0.044119	0.02994	1.376	0.169	1.04205 (0.983 – 1.105)
**Glucose**	0.00528	0.00237	2.230	**0.026**	1.00529 (1.001 – 1.010)
**Heart Rate**	-0.00569	0.00916	-0.622	0.534	0.99432 (0.977 – 1.012)

Estimates represent the log odds of “groups = 0 control” vs. “groups = 1 ACS”.
B, Regression coefficient; SE, Standard error; Z, Wald test statistic; OR, Odds ratio; CI, Confidence interval; ACS, Acute coronary syndrome; SBP, Systolic blood pressure; BMI, Body mass index.
