# Supplementary material for: Investigation of Prothrombin G20210A and Factor V Leiden G1691A Variants in Patients with Acute Coronary Syndrome Presenting to the Emergency Department with Chest Pain
Source: Genes (Basel). 2025 Dec 12;16(12):1490. doi: 10.3390/genes16121490 (PMC12733124; doi:10.3390/genes16121490)
Supplement: Supplementary file 1 [file genes-16-01490-s001.zip › Supplement Table S2.pdf]

Binomial Logistic Regression Model 3

Model Fit Measures

				Overall Model Test		
**Model**	**Deviance**	**AIC**	**R^2N**	**χ^2**	**df**	***p***
1	201	229	0.528	115	13	**<.001**

Models estimated using sample size of N = 231.

Model Coefficients – groups

					95% Confidence Interval
**Predictor**	**B**	**SE**	**Z**	***p* value**	**OR (95% CI)**
**Intercept**	4.52700	9.73427	0.465	0.642	92.481 (4.797– 1.791)
**Age**	0.03020	0.01190	2.537	**0.011**	1.031 (1.007 – 1.05)
**rs1799963 (GG/GA +AA)**	1.82779	0.92147	1.984	**0.047**	6.220 (1.022 – 37.86)
**rs6025 (GG/GA +AA)**	0.67032	0.53609	1.250	0.211	1.955 (0.684 – 5.59)
**Sex (Male– Female)**	0.14854	0.37761	0.393	0.694	1.160 (0.553 – 2.43)
**SBP**	-0.00430	0.00805	-0.534	0.594	0.996 (0.980 – 1.01)
**BMI**	0.07906	0.03900	2.027	**0.043**	1.082 (1.003 – 1.17)
**Glucose**	0.00193	0.00310	0.623	0.534	1.002 (0.996 – 1.01)
**Heart Rate**	-0.00766	0.01110	-0.690	0.490	0.992 (0.971 – 1.01)
**Potassium**	-0.25854	0.35152	-0.735	0.462	0.772 (0.388 – 1.54)
**Sodium**	-0.07494	0.06989	-1.072	0.284	0.928 (0.809 – 1.06)
**ALT**	-0.00105	0.00749	-0.140	0.889	0.999 (0.984 – 1.01)

**AST**	0.01204	0.01048	1.149	0.250	1.012 (0.992 – 1.03)
**Troponin I**	2.47730	0.38180	6.489	**<.001**	11.909 (5.635 – 25.17)

Estimates represent the log odds of “groups = 0 control” vs. “groups = 1 ACS”.
B, Regression coefficient; SE, Standard error; Z, Wald test statistic; OR, Odds ratio; CI, Confidence interval; ACS, Acute coronary syndrome; SBP, Systolic blood pressure; BMI, Body mass index; ALT, Alanine aminotransferase; AST, Aspartate Aminotransferase
